# Clinician-Created Educational Video Resources for Shared Decision-making in the Outpatient Management of Chronic Disease: Development and Evaluation Study

**DOI:** 10.2196/26732

**Published:** 2021-10-11

**Authors:** Joshua G Kovoor, Daniel McIntyre, William W B Chik, Clara K Chow, Aravinda Thiagalingam

**Affiliations:** 1 University of Adelaide The Queen Elizabeth Hospital Adelaide Australia; 2 Westmead Applied Research Centre Faculty of Medicine and Health University of Sydney Sydney Australia; 3 Department of Cardiology Westmead Hospital Sydney Australia

**Keywords:** Shared decision-making, chronic disease, outpatients, audiovisual aids, atrial fibrillation, educational technology, teaching materials, referral and consultation, physician-patient relations, physicians

## Abstract

**Background:**

The provision of reliable patient education is essential for shared decision-making. However, many clinicians are reluctant to use commonly available resources, as they are generic and may contain information of insufficient quality. Clinician-created educational materials, accessed during the waiting time prior to consultation, can potentially benefit clinical practice if developed in a time- and resource-efficient manner.

**Objective:**

The aim of this study is to evaluate the utility of educational videos in improving patient decision-making, as well as consultation satisfaction and anxiety, within the outpatient management of chronic disease (represented by atrial fibrillation). The approach involves clinicians creating audiovisual patient education in a time- and resource-efficient manner for opportunistic delivery, using mobile smart devices with internet access, during waiting time before consultation.

**Methods:**

We implemented this educational approach in outpatient clinics and collected patient responses through an electronic survey. The educational module was a web-based combination of 4 short videos viewed sequentially, followed by a patient experience survey using 5-point Likert scales and 0-100 visual analogue scales. The clinician developed the audiovisual module over a 2-day span while performing usual clinical tasks, using existing hardware and software resources (laptop and tablet). Patients presenting for the outpatient management of atrial fibrillation accessed the module during waiting time before their consultation using either a URL or Quick Response (QR) code on a provided tablet or their own mobile smart devices. The primary outcome of the study was the module’s utility in improving patient decision-making ability, as measured on a 0-100 visual analogue scale. Secondary outcomes were the level of patient satisfaction with the videos, measured with 5-point Likert scales, in addition to the patient’s value for clinician narration and the module’s utility in improving anxiety and long-term treatment adherence, as represented on 0-100 visual analogue scales.

**Results:**

This study enrolled 116 patients presenting for the outpatient management of atrial fibrillation. The proportion of responses that were “very satisfied” with the educational video content across the 4 videos ranged from 93% (86/92) to 96.3% (104/108) and this was between 98% (90/92) and 99.1% (107/108) for “satisfied” or “very satisfied.” There were no reports of dissatisfaction for the first 3 videos, and only 1% (1/92) of responders reported dissatisfaction for the fourth video. The median reported scores (on 0-100 visual analogue scales) were 90 (IQR 82.5-97) for improving patient decision-making, 89 (IQR 81-95) for reducing consultation anxiety, 90 (IQR 81-97) for improving treatment adherence, and 82 (IQR 70-90) for the clinician’s narration adding benefit to the patient experience.

**Conclusions:**

Clinician-created educational videos for chronic disease management resulted in improvements in patient-reported informed decision-making ability and expected long-term treatment adherence, as well as anxiety reduction. This form of patient education was also time efficient as it used the sunk time cost of waiting time to provide education without requiring additional clinician input.

## Introduction

Chronic disease is the leading cause of disease burden and mortality worldwide, with increasing prevalence due to an aging global population [1]. Cardiovascular disease is one of the major categories of chronic disease, and atrial fibrillation (AF) is widely recognized as one of the most common chronic conditions [2].

Ongoing outpatient consultations are an essential component of chronic disease management [3], and one strategy that could have considerable utility in this setting is that of “shared decision-making” [4]. Shared decision-making involves bidirectional information exchange within the clinician-patient relationship before making management decisions [5], and can optimize the practice of evidence-based medicine [6]. Patients are informed and care is patient-centered as the patient is empowered to communicate their personal values and management preferences for the clinician to individualize suggested management options [7]. A patient’s level of involvement in shared decision-making is influenced by their level of health literacy [8], and accordingly educational decision aids are facilitative [9]. Decision aids have shown potential benefit [10] when implemented while patients are waiting for the consultation [11].

Audiovisual education aids, including videos, can be an effective method of improving patient health literacy [10,12,13]. However, clinicians may be reluctant to use those that are commonly available (eg, those publicly available on health care websites or YouTube) if they are generic or contain information of insufficient accuracy, quality, or currency [14]. Patient care may benefit from clinicians creating their own audiovisual content and delivering it to their patients within the clinical setting.

Based on findings from the prior literature [15], we hypothesized that clinician-created audiovisual content could be created with limited resources and would be acceptable and improve the ability of patients to contribute to the decision-making process. Integral to this is understanding effects of the intervention on anxiety around consultation (“white coat” effects) [16] and potential long-term compliance to formulated management plans [17]. We aimed to evaluate this approach within outpatient management of chronic disease (represented by AF in this instance [2]), whereby clinicians create audiovisual patient education in a time- and resource-efficient manner for opportunistic delivery, using mobile smart devices with internet access, during waiting time before consultation. Specifically, we aimed to assess the following: patient satisfaction with the individual videos and the approach overall, patient response to clinician narration within the content, and the patient-perceived effect of the approach on patient decision-making, patient anxiety around consultation, and potential long-term treatment adherence.

## Methods

### Study Design

We conducted a prospective, nonrandomized, observational study of 116 patients presenting specifically for the outpatient management of AF. Patients provided informed consent through a validated electronic form before commencing the audiovisual module on a smart device. Ethical approval for the study was obtained from the Western Sydney Local Health Network Human Research Ethics Committee (item number 2011–18).

### Setting and Participants

Consecutive patients presenting for outpatient management of AF were prospectively recruited at a specialist outpatient clinic within a large university teaching hospital in Sydney, Australia. The site was within a public metropolitan hospital that serves patients of the Western Sydney Local Health District. As of 2018, the district provides AUD $1.8 billion (US $1.3 billion) in public health care to over 120 suburbs consisting of a demographically diverse population; approximately 50% of the residents have chronic conditions [18]. Potential participants were identified by study personnel via screening of the specialist outpatient clinic lists.

Patients were eligible for inclusion if they had presented to the specialist outpatient clinic specifically for consultation for the management of AF, and were English speaking, willing and able to use smart devices, and could provide informed consent. Patients were excluded if they were unable to speak English, refused consent, or had visual impairment or any other factor that prevented them from using the provided tablet (eg, rheumatoid arthritis). All prospective patients who met the inclusion criteria were enrolled in the study.

### Development of the Audiovisual Module

The educational audiovisual module was designed to be a web-based combination of 4 short videos (privately hosted on YouTube) to be viewed sequentially, accompanied by questions gauging patient experience. The module was consolidated using Research Electronic Data Capture (REDCap) [19]. Data from the module were collected and coded on REDCap via a secure database.

The audiovisual module sought to provide a baseline level of disease-specific health literacy to patients that supplemented information provision and improved shared decision-making in the subsequent outpatient consultation. For this study, two of the authors (AT and JGK) consulted the latest guidelines and peer-reviewed literature, and decided on an up-to-date, reliable, yet simple syllabus covering the fundamental concepts of the pathophysiology, clinical presentation, diagnosis, and management of AF. For this, we searched for studies of any design, in any setting, within Scopus using the search term “atrial fibrillation” in all fields. To ensure that the most prominent recent AF literature was reviewed, the search was date restricted from January 2016 to December 2018, and the 46,393 resultant records were sorted in order of total number of citations. From the prominent recent AF literature that was reviewed, the two most cited guidance statements specifically relevant to the management of AF [20,21] were discussed by two authors (AT and JGK) and concepts and the syllabus outline were subsequently formulated by consensus. The resultant module contained four succinct videos encompassing the following topics: (1) “What is AF?” (2) “AF Management,” (3) “Stroke risk and anticoagulation,” and (4) “Lifestyle modification.”

**Figure 1:**
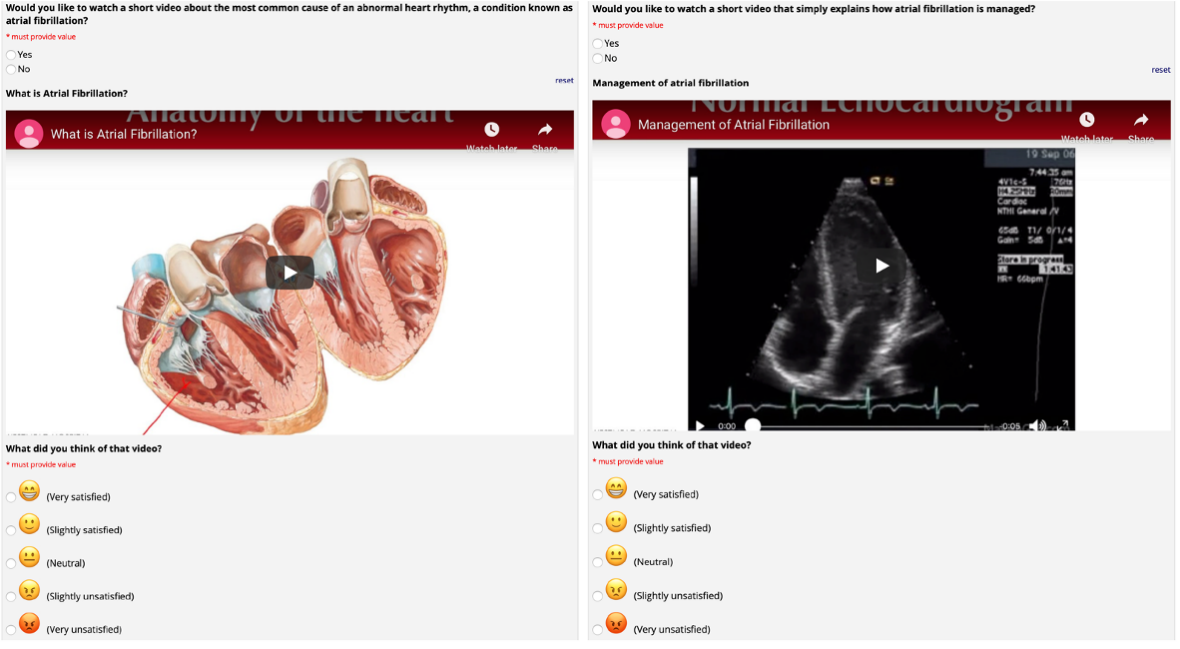
A sample of the web-based audiovisual educational module consolidated using REDCap.

For the measurement of outcomes, we used Likert scales and visual analogue scales, which have both demonstrated reliability and validity as health measurement tools [22]. The level of patient satisfaction for each individual video and satisfaction with the videos overall were recorded on 5-point Likert scales [23]. Further, 0-100 visual analogue scales were used to gauge the patient’s response to clinician narration, as well as the module’s patient-perceived utility in improving patient decision-making, anxiety around consultation, and potential long-term treatment adherence. The visual analogue scales and Likert scales were created and combined with the 4 videos for the audiovisual module using REDCap (Figure 1).

After the simple syllabus was formulated, the clinician (AT) developed the audiovisual module over a 2-day span between clinical commitments and within normal working hours (approximately 3 hours total). No disruption to regular clinical duties was experienced. The only material resources used in the process were that of a laptop containing Microsoft PowerPoint (Microsoft Corp) and an Apple iPad (Apple Inc), both of which were already owned by the clinician, thus not incurring any additional financial costs.

**Figure 2:**
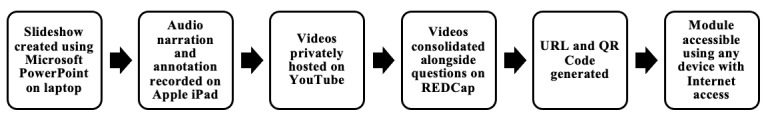
Technique for audiovisual module creation by a clinician.

To make the 4 educational videos within the module, the clinician created 4 brief slideshows using PowerPoint on a laptop, then recorded audio narration simultaneously to annotate the slideshows using the Apple iPad using the Screen Recording function available by default on iOS 13 or later (Figure 2). The latter was done in a single take; on average, it took the clinician 3 minutes and 42 seconds to record the audio narration and annotation that complemented the slideshows within each individual video. The videos were subsequently privately hosted on YouTube in order to be consolidated alongside the patient experience survey on REDCap. This ensured that no advertising material was delivered with the videos by the YouTube website. REDCap then generated a URL, also known as a web address, and a Quick Response (QR) code, both of which enabled access to the educational module using any device with internet access.

All information provided within the module was only included following brief inspection of the latest peer-reviewed literature on the associated topics by the clinician to update existing knowledge. Further, the module was recorded in English with language and readability aimed below an eighth grade level to enhance accessibility for the general adult population [24]. The audiovisual module, along with all included images and visual media, was consolidated solely for private use limited to the duration of the study. It was not marketed or sold for commercial purposes.

### Delivery During Waiting Time

To minimize potential bias attributable to the halo effect [25], patients were approached by members of staff in the specialist clinic other than the clinician who created the educational videos. Following informed consent, patients completed the audiovisual educational module during the otherwise nonclinically utilized waiting time before their consultation for AF. Patients accessed the web-based module through either the URL or QR code generated by REDCap. This was done using either a tablet that was already owned by the treating specialist in the clinic or mobile smart devices that the patients already owned.

The entire audiovisual module was designed to take a maximum of 20 minutes for the patient to complete, so as to comfortably be completed during the expected waiting time before outpatient consultation [26]. Further, the cumulative duration of all four videos totaled 14 minutes and 46 seconds, so as to maximize concentration and minimize the chances of attention decline [27].

### Data Elements

The primary outcome of the study was the audiovisual educational module’s utility in improving patient decision-making ability during the subsequent outpatient consultation [17], which was measured in an anonymous manner using a 0-100 visual analogue scale. The secondary outcomes were the level of patient satisfaction for each video and satisfaction with the videos overall (both measured using 5-point Likert scales), as well as the patient’s value for clinician narration, and the module’s patient-perceived utility in improving anxiety around consultation and potential long-term treatment adherence (all measured using 0-100 visual analogue scales in an anonymous manner). Data were extracted by two authors (JGK and AT) from the secure REDCap database as a CSV file.

### Statistical Analysis

Data were assessed in Python (open source, Python Software Foundation) using the Pandas library (version 1.0.4, open source, PyData), with results reported using medians and interquartile ranges. Figures were prepared using the Plotly (version 4.8.1) library. Friedman test and post hoc analysis were performed using Statsmodels (version 0.10.2, open source) as the data were not normally distributed, with repeated observations on the same individuals.

## Results

### Overview

Between January 2019 and August 2019, all 116 prospective patients who met the aforementioned inclusion criteria were enrolled in the study. Response rates for the 5-point Likert scales progressively decreased with each successive video, with 93.1% (108/116) of the total cohort conveying their level of satisfaction with the first video (“What is AF?”), 87.1% (101/116) for the second (“AF Management”), 85.3% (99/116) for the third (“Stroke risk and anticoagulation”), and 79.3% (92/116) for the fourth (“Lifestyle modification”). In addition, 85.3% (99/116) of the total cohort reported the effect of the audiovisual module on consultation anxiety, decision-making ability, and likelihood of potential treatment adherence through the 0-100 visual analogue scales, with 84.5% (98/116) reporting the importance of clinician narration.

### Likert Scales

**Figure 3:**
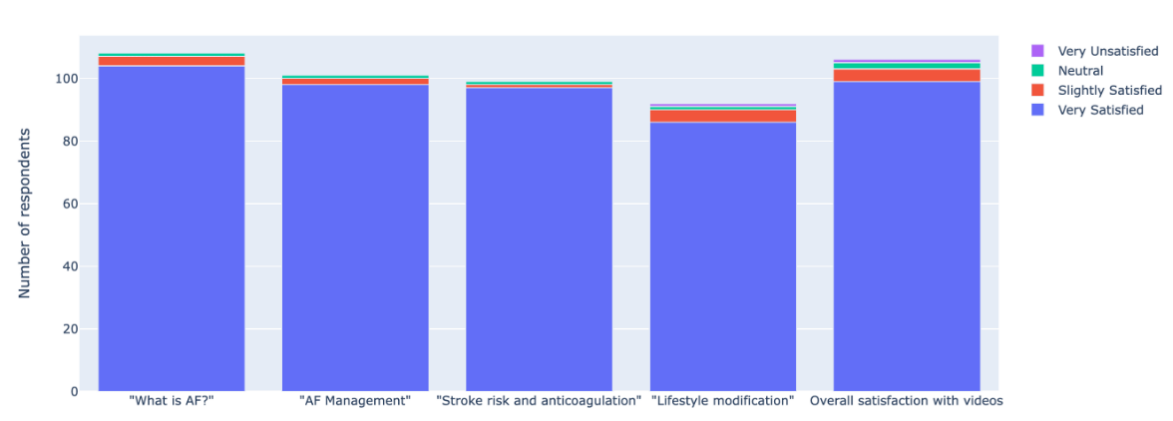
Patient satisfaction with clinician-created videos. AF: atrial fibrillation.

The proportion of participants that were “very satisfied” with the educational video content ranged from 93% (86/92) to 96.3% (104/108) for the individual videos. In the final 5-point Likert scale assessing overall satisfaction with all 4 videos, 93.4% (99/106) of responders were “very satisfied,” with 0.9% (1/106) reporting dissatisfaction. For the first video (“What is AF?”), 99.1% (107/108) of responders reported satisfaction with the content, compared to 99.0% (100/101) for the second (“AF Management”), 99.0% (98/99) for the third (“Stroke risk and anticoagulation”), and 98% (90/92) for the fourth (“Lifestyle modification”). There were no reports of dissatisfaction for the first 3 videos, and 1% (1/92) of responders reported dissatisfaction for the fourth video. Patient satisfaction with the clinician-created educational videos, as obtained through 5-point Likert scales, is represented in Figure 3.

### Visual Analogue Scales

**Figure 4:**
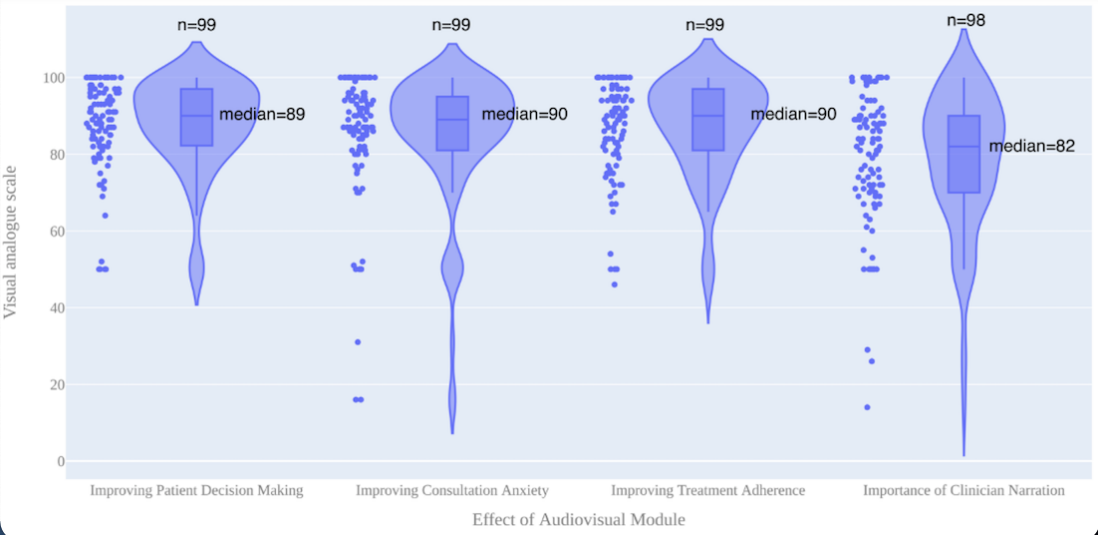
Patient responses to the audiovisual module.

Median scores on the four 0-100 visual analogue scales ranged from 82 to 90. The audiovisual module resulted in median reported patient-perceived scores of 90 (IQR 82.5-97) for improving patient decision-making, 89 (IQR 81-95) for improving consultation anxiety, 90 (IQR 81-97) for improving potential treatment adherence, and 82 (IQR 70-90) for the clinician’s narration adding benefit to the patient experience. Patient responses to the 0-100 visual analogue scales are presented in Figure 4.

Friedman test results yielded a Friedman statistic value of 33.3 (*P*<.001). The Nemenyi post hoc analysis showed that only “Importance of clinician narration” was significantly different (*P*=.001) from all the other evaluations. The other evaluations did not differ significantly from each other. These results are presented in Table 1.

**Table 1:** Analysis of patient responses using the Friedman test.

Evaluation	Improving consultation anxiety	Improving patient decision making	Improving potential treatment adherence	Importance of clinician narration
Improving consultation anxiety	1	.70	.90	.001
Improving patient decision making	.70	1	.49	.001
Improving potential treatment adherence	.90	.49	1	.001
Importance of clinician narration	.001	.001	.001	1

## Discussion

### Principal Findings

A clinician-created audiovisual patient education module with content focusing on improving consultation efficiency and shared decision-making received scores indicating a high level of acceptability and patient-perceived utility. The video content was developed by a clinician using electronic resources that are relatively common in the developed world, during interstitial time between clinical duties, and was delivered using mobile smart devices while patients were waiting for their appointments. The majority of participants perceived the clinician’s creation and narration of the module to be positive and effective in delivering the educational content of the disease-specific videos.

Shared decision-making provides a potential solution for achieving adequate efficiency while improving patient autonomy [28]; however, this may not translate to clinical practice if the appropriate approach to implementation is not taken [29]. An element of usefulness in our approach is that it not only improves shared decision-making, but also uses waiting time (sunk time cost) for an improved patient experience that does not add to the overall length of the consultation. Further, our approach demonstrates that clinicians can use off-the-shelf tools to simply create their own highly customized educational content tailored specifically for their patients. Further, it is implementable with minimal additional resources.

Patient education has been shown to be more effective when delivered in an audiovisual format than when presented solely through visual pamphlets or verbal one-to-one dialogue [30]. However, clinical utility for the management of chronic disease is limited when audiovisual patient education has required additional outpatient appointments [31]. This intervention used shorter duration, highly focused videos, allowing delivery in the waiting room so that patients would not have to make additional visits. Increasing access to unreliable, inaccurate, and outdated sources via the internet can increase confusion and cause difficulties during consultations for the management of patients’ conditions [32]. This can potentially be reduced through the provision of up-to-date and reliable information directly from a patient’s own treating clinician, as a supplement to scheduled consultations. Our approach allows for the provision of reliable health education in a manner that is streamlined for integration within both the clinician (through time and resource efficiency) and patient (through tailored, accessible education) experience of outpatient consultation.

The audiovisual educational module demonstrated utility in improving patient-reported decision-making ability for the subsequent outpatient consultation for AF management. However, due to a lack of any of the validated outcome measures or objective endpoints that are found within the existing literature of studies investigating shared decision-making in AF management [33], comparison between previous studies and our study is compromised. The high levels of patient satisfaction with both the videos and clinician narration suggest that there may be potential usefulness in clinician-created audiovisual educational content for the management of chronic disease. Further, anxiety [34], particularly that associated with the “white coat” effect [16], and long-term treatment adherence [17,35] have a significant impact on a patient’s decision-making ability regarding the management of any chronic disease. Accordingly, our positive findings imply potential value in these domains. Our finding of patient-reported improvements to potential treatment adherence is in line with the existing literature, which has demonstrated the efficacy of video-assisted patient education in positively modifying the behaviors of patients with chronic disease if designed and delivered correctly [36].

There were limitations to this study that require future evaluation. Our study did not have a control group that enabled comparison; the study was observational and baseline characteristics of the participants were not collected. However, we prospectively included a consecutive series of eligible patients to limit patient bias, and all patients answered the same questionnaire. Our study was limited by subjective responses from patients as outcome measures, instead of outcome measures such as recurrence of AF or occurrence of its complications. The data collected referred specifically to the patients’ own perceptions, which may carry inherent bias. We did not collect data using validated measures of patient-reported experience or health literacy. Further, we delivered the educational module only in English, and excluded non–English-speaking patients. Within the literature search that informed the curriculum for our educational module, sorting search results in Scopus by total numbers of citations identified the most prominent articles within the search time frame, but added a source of bias regarding the data informing our intervention. As our study was conducted in 2019, any evidence published since then has not been integrated. Further, only one database was searched prior to the development of our curriculum, so some literature may have been missed. The study was limited to a single center, so this approach’s multicenter applicability has not been tested. Additionally, although the clinician in our study was able to carry out our approach in a time- and resource-efficient manner, this approach may not translate to certain doctors with different levels of resources, time within schedules, and technology skills.

Findings from this pilot study may be useful for future research in this area. Although audio narration was used in this study, future studies may benefit from the inclusion of video of the treating clinician and investigation of subsequent effects on patient trust, anxiety reduction, and potential adherence. Clinical interactions within settings outside the management of chronic disease at outpatient clinics may also benefit from this approach and should be investigated. Usefulness of the approach relative to the socioeconomic status of the health care provider may be important to delineate for the maximization of global scalability. Further, exploration of utility for health professionals in other areas of medicine, surgery, nursing, and allied health ought to be explored. Study design in this future research can be improved by incorporating a comparison (ideally with randomization) against other forms of patient education as well as more concrete endpoints, such as objective health metrics or validated scoring schemas. The measures of patient perceptions that were recorded in this pilot study may be better assessed via a longitudinal approach that facilitates the evaluation of temporal trends. Explanations for changes in patient perceptions throughout the course of the videos may have greater clarity if more qualitative data points are also collected in addition to measures of satisfaction.

### Conclusions

This approach to outpatient consultation for the management of chronic disease may provide benefit for shared decision-making between clinicians and patients, overall leading to the improvement of care while maintaining a patient-centered focus. It benefits clinicians by combining their individual content knowledge with technology to create highly customized disease-specific audiovisual educational material for their patients. It benefits patients by allowing them to learn about their condition in a nonconfrontational situation without additional investment of time or effort. Patient satisfaction may be improved by converting a potentially negative situation (the sunk time cost of waiting for a medical appointment) to a positive experience. This pilot study demonstrated the potential utility of this approach in a specific setting; however, it may have widespread applicability across a large number of clinical scenarios, including those outside the management of chronic disease and outside the outpatient setting. Future research should explore this potential widespread applicability of our approach through studies of larger size and strong design.

## Abbreviations

AF: atrial fibrillation
QR: Quick Response
REDCap: Research Electronic Data Capture
